# Perception of Emotion and Postural Stability Control at Different Distances

**DOI:** 10.16910/jemr.15.4.6

**Published:** 2022-11-12

**Authors:** Soufien Chikh, Salma Charrada, Eric Watelain

**Affiliations:** University of Sfax, Research Laboratory EM2S, LR19JS01, High Institute of Sport and Physical Education of Sfax.; Université de Toulon, Laboratoire UR IAPS 201723207F, 83041, Toulon, France

**Keywords:** Emotion, distance, vision, valence–arousal, postural control, equilibrium

## Abstract

The effect of emotion on postural control has been widely demonstrated in the literature. Postural control also depends on the distance that separates the subject from the observed stimulus. This work examines (i) the effect of distance on the perception of emotional stimuli and (ii) its effect on postural control. Sixty-eight women were asked to maintain orthostatic equilibrium under three emotional conditions (positive, negative, and neutral) at four distances (0.5 m, 2.1 m, 6 m, and 10 m). The findings showed that the perception of emotions was not influenced by distance but was influenced by valence and intensity, and that postural control was not influenced by emotional valence but by distance, with reduced oscillation amplitudes at 0.5 m distance. The perception of the image (valence and intensity) depended on the content, but not on the distance, and the presentation of emotional images tended to activate the defensive system, regardless of the emotional content. The center of pressure sway amplitude increased with an eye–object distance of up to 6 m (role of vision). The perception of the emotional effect was not linked to the distance effect on the postural control of women in static positions.

## Introduction

Postural control (PC) is essential when performing most everyday activities and allows for effective interaction with the environment (43). Postural stability (PS) is the inherent capacity of a person to maintain, reach, and restore a specific state of equilibrium to prevent falling (36).

Considerable evidence suggests that one’s emotional state can influence PC during locomotion (11; 32; 21), walking initiation ( 31; 45), and even in the orthostatic position (17; 3; 14; 15; 22). The motivational component, or approach-avoidance behavior, is influenced by emotions and depends on valence (level of pleasure) (17). Approach behavior is characterized by a decrease in the distance between the subject and the stimulus and is favored by positive stimuli (activation of the appetitive system). Conversely, avoidance behavior is characterized by an increase in distance and is favored by negative stimuli (activation of the defensive system) (23; 17; 22).

Many theories of emotions postulate a basic association between emotions and certain types of behavior, such as approach and avoidance (e.g., Frijda, 1986). It is generally believed that emotions are divided into two distinct motivational systems that enable an organism to respond effectively to emotionally relevant stimuli in the environment (Lang et al., 1990). A defensive motivational system would cause avoidance behavior away from unpleasant, negative stimuli (which results in an increase in distance between the subject and the stimulus), whereas an appetitive motivational system is hypothesized to encourage the organism to approach pleasant, positive stimuli (which results in a decrease in the distance between the subject and the stimulus).

Recent theories have emphasized the important role of emotion in motor control (26). For two decades, the number of related papers has exponentially increased, placing these links at the core of new research questions that have yet to provide the answers necessary for understanding these mechanisms. Understanding the relationship between emotion and behavior is essential and represents a major factor in acquiring, controlling, and developing motor skills. The effect of emotion on PC was first investigated through tasks for maintaining the posture-kinetic position using images, such as those of the International Affective Picture System (IAPS©) (17; 3; 39). Several studies have analyzed the effect of valence (37; 15) and arousal (i.e., the intensity of the image, with intensity seen in both negative and positive images) on postural equilibrium (18). Postural sway (the movement of the center of mass in a standing position) is considerably reduced during unpleasant images (3). When participants were faced with unpleasant images compared to neutral or pleasant images, “freezing” behavior (i.e., a decrease in the amplitude and displacement of the center of pressure [CoP]) was often observed (3). While some authors believe that changes in postural responses are linked to the valence of emotional content (8; 3; 14; 37; 15), others claim that arousal influences postural sway in the sagittal plane (18; 22; 7).

These adaptive postural responses contribute to regulating the distance between the subject and the emotional stimulus. However, none of the studies aiming to understand the effect of emotional manipulation on behavior have considered the effect of the physical distance between the subject and the emotional stimulus. For example, an avoidance behavior may be more exacerbated if the subject is near the stimulus and therefore represents increased danger. The perception of the valence and arousal of an emotional stimulus depends on the proximal or distal distance. The proximity of unpleasant stimuli intensifies the emotions perceived compared to those that are distant (29). Mühlberger et al. (29) showed that the emotional valence of unpleasant stimuli approaching the participant was more negative compared to static or receding unpleasant stimuli. This effect of movement direction was observed only for unpleasant stimuli (not for neutral or pleasant stimuli). Neural systems involving a defensive approach are also assumed to be organized regarding distance. Proximal rewards tend to be valued more positively than distal rewards (27). At this level, spatial signals influence the degree of emotional distress and make responses more adaptive. In the literature, distance is considered an organizing notion, whether the stimulus is initially negative or not, and whether it moves in space (toward or away from “here”), in time (toward or away from “now”), or in probability (toward or away from “sure”) (19). Physical distance is not only that which separates two or more points in space (objects, persons, etc.), it also plays the role of a landmark that influences personal and affective judgments. Mühlberger et al. (29) and Davis et al. (12) showed that participants signal a significantly higher degree of valence and arousal when they are in proximity to unpleasant visual stimuli. By contrast, Valdés-Conroy et al. (42) indicated that objects with a positive valence tend to be perceived as reachable when they are outside the peripersonal space. In line with these findings, Davis et al. (12) showed that the subjective assessment of a context becomes more positive and exciting when it is proximal. These results are consistent with the theory of the motivational approach to emotion, which states that changes in spatial distance significantly influence affective responses.

The importance of vision in postural equilibrium has long been known. Indeed, better PS was found in subjects whose eyes were open compared to those whose eyes were closed (20). The eye–target distance is considered the main factor influencing this relationship of distance–PS coupling (5; 33, 34). Using galvanic vestibular stimulation (GVS), Aoki et al. (2) demonstrated that asking participants to gaze at a nearby object (small eye–object distance) reduced body sway in the mediolateral direction. Stoffregen et al. (41) observed that the amplitude of standing body sway was reduced when participants looked at nearby targets (compared to sway during the viewing of distant targets), and that postural sway was reduced during a difficult visual task (a visual search of target letters in a text) compared to sway during a less difficult visual task (viewing a blank target). We argue that the search task placed more restrictive constraints on the visual system and that postural sway was reduced to facilitate the visual search. Stoffregen et al. (41) explained that the search task placed more restrictive constraints on the visual system. In addition, postural sway was reduced to facilitate the visual search.

The results obtained by Lê and Kapoula (25) supported those of Paulus et al. (34), who claimed that proximity decreased the CoP area, the standard deviation of the AP swing, and the velocity variance. They tested the differential effects of retinal target displacement, changing size, and changing disparity in the control of anterior–posterior and lateral body sway. Retinal slips (visual movement), which are produced by postural oscillations, are detected by the central nervous system, which triggers corrective postural oscillations to stabilize posture. The detection of retinal slips by the visual system depends on the viewing distance. A decrease in distance increases the angular size of the retinal slip induced by body sway and makes it easier to detect (25).

The purpose of the present study was to examine the effect of the distance between an individual and visual stimuli on both emotion perception and orthostatic PC. It was hypothesized that proximity could exacerbate the perception of the valence and intensity of emotional content, which would later influence static postural equilibrium. Given that emotional images are perceived as having more valence and intensity with a proximal distance, the amplitudes of the CoP’s displacement are smaller for the proximal distance. We expected to observe more incidences of approach behavior for positive images and more avoidance behavior for negative images at a proximal distance.

## Methods

In the Method section, you should describe the details of how the study was conducted. You should provide the reader with enough information to be able to replicate your study. Details that are not important for replication should not be included (e.g., what type of pencils the participants used, etc.). The reader should also be able to evaluate the appropriateness of your methods for the hypothesis you made. Method sections may vary in the number of sections the authors include, but the most common sections are described below. The entire Method section should be written in past verb tense. You can use a table to report important characteristics of the method or the flow of activities. An example is provided in Table 1.

### Participants

Sixty-eight female volunteers participated in the study (with a M ± SD age of 21.46 ± 1.42 years; a height of 165 ± 6 cm; and a weight of 60.9 ± 7.25 kg). The inclusion criteria were normal or corrected vision, good health, no vestibular and/or neurologic problems, and not taking any medication that may directly or indirectly affect motor skills or the management of emotions. The exclusion criteria were a deficiency or disease of the lower limbs six months before the test (fracture, joint prosthesis, surgery) that could prevent them from standing quietly. The male gender was excluded from this experiment due to anthropometric differences and differences in emotional significance between the two genders (17). All subjects signed an informed consent letter. The institutional review board approved the study, which was carried out under the recommendations of the Helsinki Convention.

### Design

*Materials:* The static stabilometric platform (PostureWin©, Techno Concept®, Cereste, France; 40 Hz frequency, 12-bits A/Dconversion) was used to record the CoP excursions in the static postural condition. A computer was used to record the CoP data, which were synchronized with the procedure for presenting emotional images. A video projector (connected by the same computer) was used to display visual stimuli on a white mat screen 0.7 m × 1.4 m in size and was organized using Pinnacle HD software 16.

*Stimuli:* The 36 images selected from the IAPS (24) comprised 12 neutral images of objects (e.g., accessories and utensils) with a valence between 4.96 and 5.17 [7004; 7006; 7010; 7041; 7050; 7059; 7080; 7090; 7150; 7175; 7233; 7235]; 12 positive images (e.g., smiling babies and families) with a valence between 7.23 and 8.35 [2045; 2058; 2071; 2150; 2160; 2216; 2347; 2352.1; 2550; 4599; 4626: 4628]; and 12 negative images (e.g., mutilation and injuries) with a valence between 1.18 and 2.08 [3010; 3030; 3060; 3068; 3069; 3071; 3130; 3131; 3168; 3051; 3015; 3150].

*Measures: Subjective measures:* The valence and arousal of each picture were measured using the 9-point self-assessment manikin (SAM) (9). Concerning the valence or pleasure dimension, SAM ranges from a smiling, happy figure to a frowning, unhappy figure. For the arousal or intensity dimension, SAM ranges from an excited, wide-eyed figure to a relaxed, sleepy figure. *Objective measures of CoP:* Mean velocity in two dimensions ( $=\frac{\sum \sqrt{}[{\left(X(n)-X(n+1)\right)}^{2}+{\left(Y(n)-Y(n+1)\right)}^{2}]}{3}$), AP amplitude (= peak anterior – peak posterior) and ML amplitude (= peak medial – peak lateral), and the mean CoP position (= mean CoP position in image window – mean CoP position in fixation cross) in the AP axis were measured. The mean CoP position was quantified using the mean position in the fixation cross for 2 s.

### Procedure

**Figure 1.**
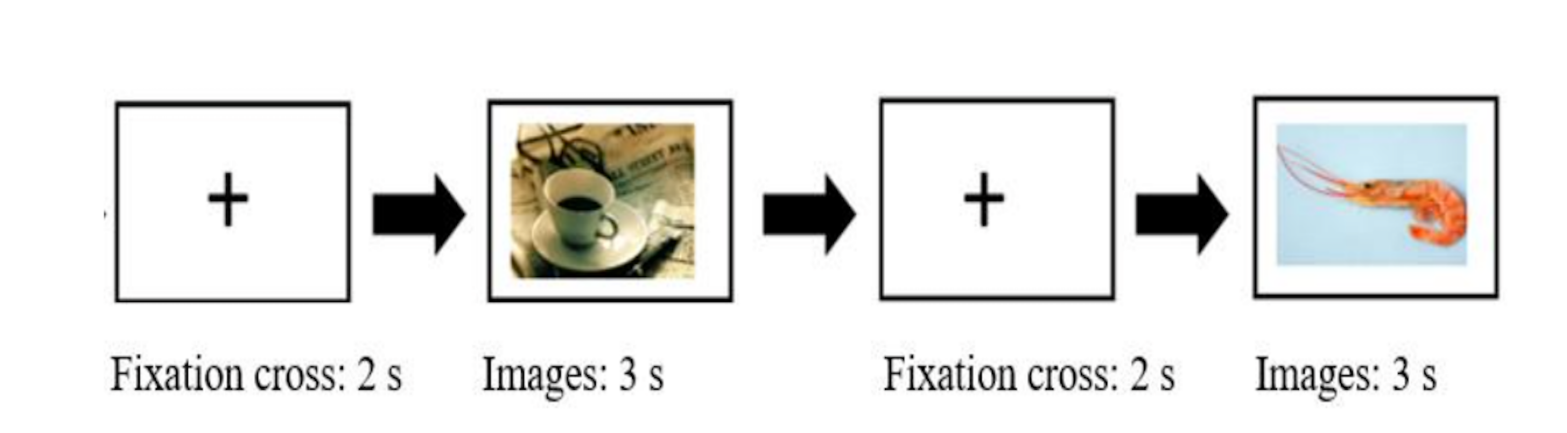
Order of presentation of images for each distance

The experiment comprised the blocked presentation of nine images (three positive, three negative, and three neutral) for each distance (0.5 m, 2.1 m, 6 m, and 10 m), resulting in 36 images. Each participant completed a familiarization session with three passages. The image order and distances were randomized and counterbalanced across all subjects. The participants stood upright on the stabilometric platform, in line with the recommendations of the French Association of Posturography (4). The participants’ feet were oriented at an angle of 30°, with heels spaced 5 cm apart. The participants were allowed to move their feet off the plate during seated breaks. To standardize the position of the feet for all measurements, the foot placement was marked on paper and placed on the stabilometric platform. Each image was presented with the same size (54 cm × 75 cm) for all distances over 3 s (i.e., 14; 37; 40), preceded by a 2 s fixation cross (Figure 1). The participants were asked to keep their equilibrium upright during the presentation of the images. Before changing the distance, each participant was asked to evaluate the valence and arousal of each image presented using the SAM scale, marked from one to nine (9). We also followed Stins and Beek’s (39) method of randomizing the emotional content of the stimuli within each scenario to obtain more adequate and selective responses and to avoid the consecutive presentation of images in the same category (35). The blocking procedure could lead to increased emotional effects over time due to increased sensitivity to images (26). The participants were given a two-minute rest in a seated position after the objective acquisition and subjective evaluation of each block to avoid fatigue.

### Statistical Analysis

The valence and arousal were evaluated using a three-emotion (positive, negative, and neutral) × four-distance (0.5 m, 2.1 m, 6 m, and 10 m) mixed-design ANOVA. The CoP parameters were evaluated using a three-emotion (positive, negative, and neutral) × four-distance (0.5 m, 2.1 m, 6 m, and 10 m) mixed-design ANOVA. Greenhouse–Geisser correction (Ɛ) was used for both ANOVAs. Tukey’s HSD was performed as a post hoc test. The statistical significance for all tests was evaluated at the 0.05 level. Partial eta squared (ηp²) values were provided only as a measure of effect size for all main effects and interactions. All statistical tests were carried out using Statistica 10 software (Statsoft©).

## Results

### Subjective Measures

Table 1 presents the subjective measures concerning the perception of valence and arousal of each emotional image of the IAPS using the 9-point SAM scale. For valence and arousal, only the effect of emotion was shown (the difference between positive, negative, and neutral images). The distance did not affect the valence or arousal scores.

**Table 1.** Summary of statistical analysis of subjective measures

	Emotion				Distance				Emotion * Distance			
	F (2, 134)	p	Ɛ	η_p_^2^	F (3, 201)	p	Ɛ	η_p_^2^	F (6, 402)	p	Ɛ	η_p_^2^
Valence	508.88	< .0001	.76	.88	.73	.47	.67	.01	.6	.62	.53	.008
Arousal	161.45	< .0001	.99	.7	.34	.78	.93	.005	.71	.6	.78	.01

**Figure 2.**
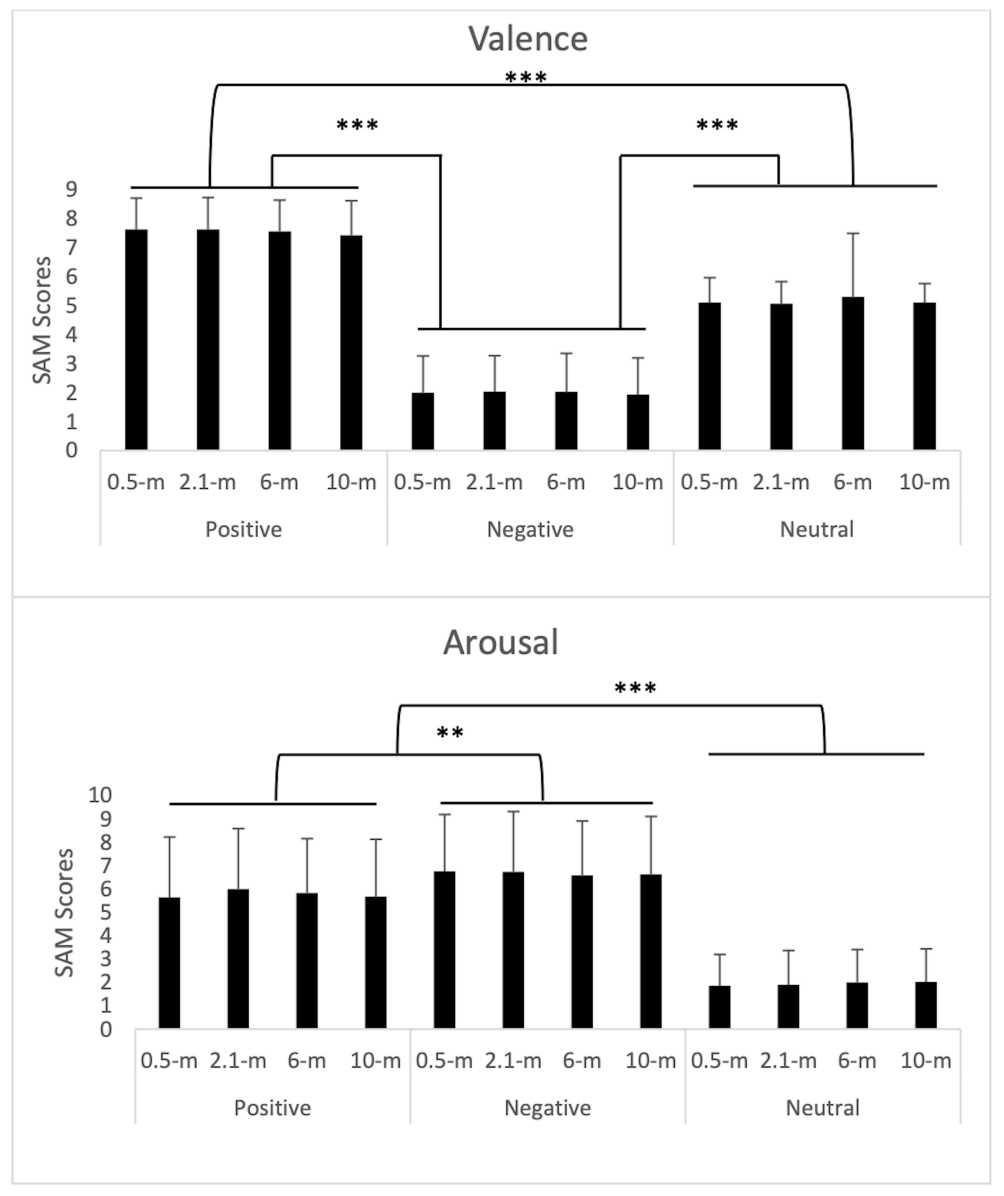
Valence and arousal scores for positives, negatives, and neutrals images. Note. SAM =Self-Assessment-Manikin; ** p< .01; *** p< .0001

The pattern of *valence scores* was different across all three categories (*p* < 0.0001), with a high score concerning positive (7.56 ± 1.11) than negative (1.99 ± 1.27) and neutral images (5.14 ± 1.27) and *p* < 0.001 between the three image categories, as shown by Tukey’s *post hoc* test (Figure 2).

With respect to *arousal*, both positive (5.77 ± 2.48) and negative (6.66 ± 2.45) images were scored as more arousing compared to neutral images (1.94 ± 1.4), with *p* < 0.0001. Negative images were also scored as more arousing compared to positive images, with *p* < 0.01, as shown by Tukey’s *post hoc* test (Figure 2).

### Objective Measures

Table 2 summarizes the statistical tests. These objective measurements were related to the CoP parameters, which were tested according to emotion, distance, and the emotion-distance interaction. The emotion factor concerns positive, negative, and neutral images, while the distance factor concerns 0.5 m, 2.1 m, 6 m, and 10 m.

**Table 2.** Summary of statistical analysis of objective measures

	Emotion				Distance				Emotion * Distance			
	F (2, 134)	p	Ɛ	η_p_^2^	F (3, 201)	p	Ɛ	η_p_^2^	F (6, 402)	p	Ɛ	η_p_^2^
Anteroposterior amplitude	.10	.89	.97	.00	15.58	<.0001	.95	.18	2.49	.29	.84	.01
Mediolateral amplitude	.27	.76	.90	.00	7.27	<.001	.95	.09	1.31	.25	.78	.01
Mean velocity in 2 dimensions	1.43	.24	.93	.02	10.32	<.0001	.85	.13	1.79	.09	.85	.02
Mean position	3.32	<.05	.95	.04	.64	.58	.97	.00	.51	.79	.82	.00

**Figure 3.**
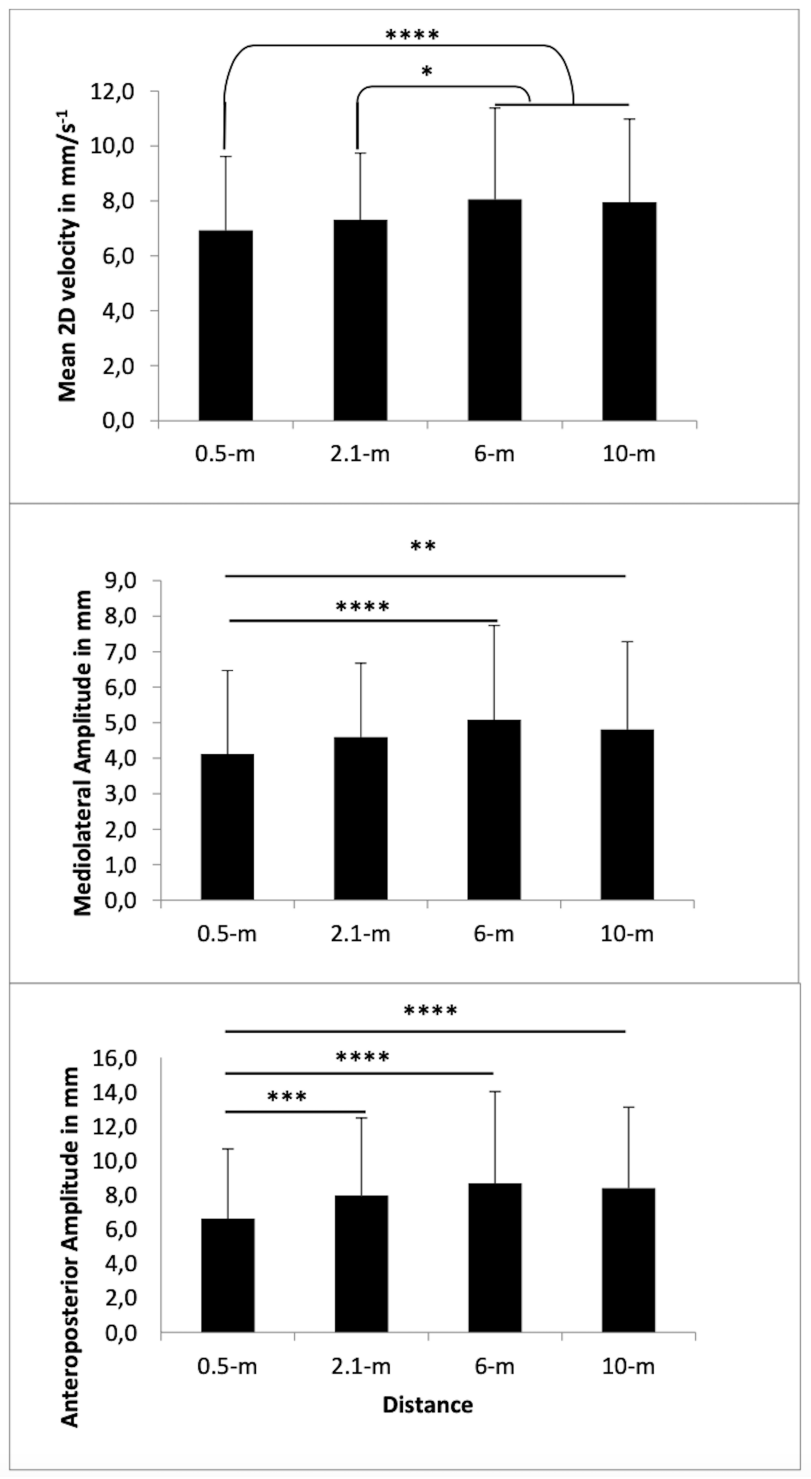
Mean velocity of the COP in 2 dimensions, mediolateral and anteroposterior amplitude according to each distance. Note. * p< .05; **p< .01; *** p< .001; **** p< .0001

The *AP amplitude* was affected only by the distance (*p* < 0.0001): according to Tukey’s *post hoc* test (Figure 3), 0.5 m showed less amplitude (6.6 ± 4 mm) than 2.1 m (8 ± 4.5 mm, *p* < 0.001), 6 m (8.7 ± 5.3 mm, *p* < 0.0001), and 10 m (8.4 ± 4.7 mm, *p* < 0.0001). No significant difference was observed between 2.1 m vs. 6 m (*p* = 0.11), 2.1 m vs*.* 10 m (*p* = 0.54), and 6 m vs*.* 10 m (*p* = 0.81). Emotion did not affect the AP amplitude (*p* = 0.89), and no difference was observed between the positive (8 ± 5 mm), negative (7.9 ± 4.6 mm), and neutral images (7.9 ± 4.4 mm).

The *ML amplitude* was affected by the distance (*p* < 0.001): according to Tukey’s *post hoc* test (Figure 3), 0.5 m showed less amplitude (4.1 ± 2.3 mm) than 2.1 m (4.6 ± 2 mm, *p* = 0.012), 6 m (5.1 ± 2.7 mm, *p* < 0.0001), and 10 m (4.8 ± 2.5 mm, *p* < 0.01). No significant difference was observed between 2.1 m vs*.* 6 m (*p* = 0.09), 2.1 m vs*.* 10 m (*p* = 0.72), and 6 m vs*.* 10 m (*p* = 0.58). Emotion did not affect the ML amplitude (*p* = 0.76), and no difference was observed between the positive (4.7 ± 2.5 mm), negative (4.7 ± 2.4 mm), and neutral images (4.6 ± 2.3 mm).

The *mean velocity of CoP in the two dimensions* was affected by the distance (*p* < 0.0001): according to Tukey’s *post hoc* test (Figure 3), 0.5 m (6.9 ± 2.7 mm) and 2.1 m (7.3 ± 2.4 mm) showed less velocity than 6 m (8.1 ± 3.3 mm, *p* < 0.0001, *p* < 0.05, respectively) and 10 m (8 ± 3 mm, *p* < 0.0001, *p* < 0.05, respectively). No significant difference was observed between 0.5 m vs. 2.1 m (*p* = 0.33) and between 6 m vs. 10 m (*p* = 0.97). Emotion did not affect 2D velocity (*p* = 0.24), and no difference was observed between the positive (7.7 ± 3.3 mm), negative (7.5 ± 2.7 mm), and neutral images (7.5 ± 2.8 mm).

**Figure 4.**
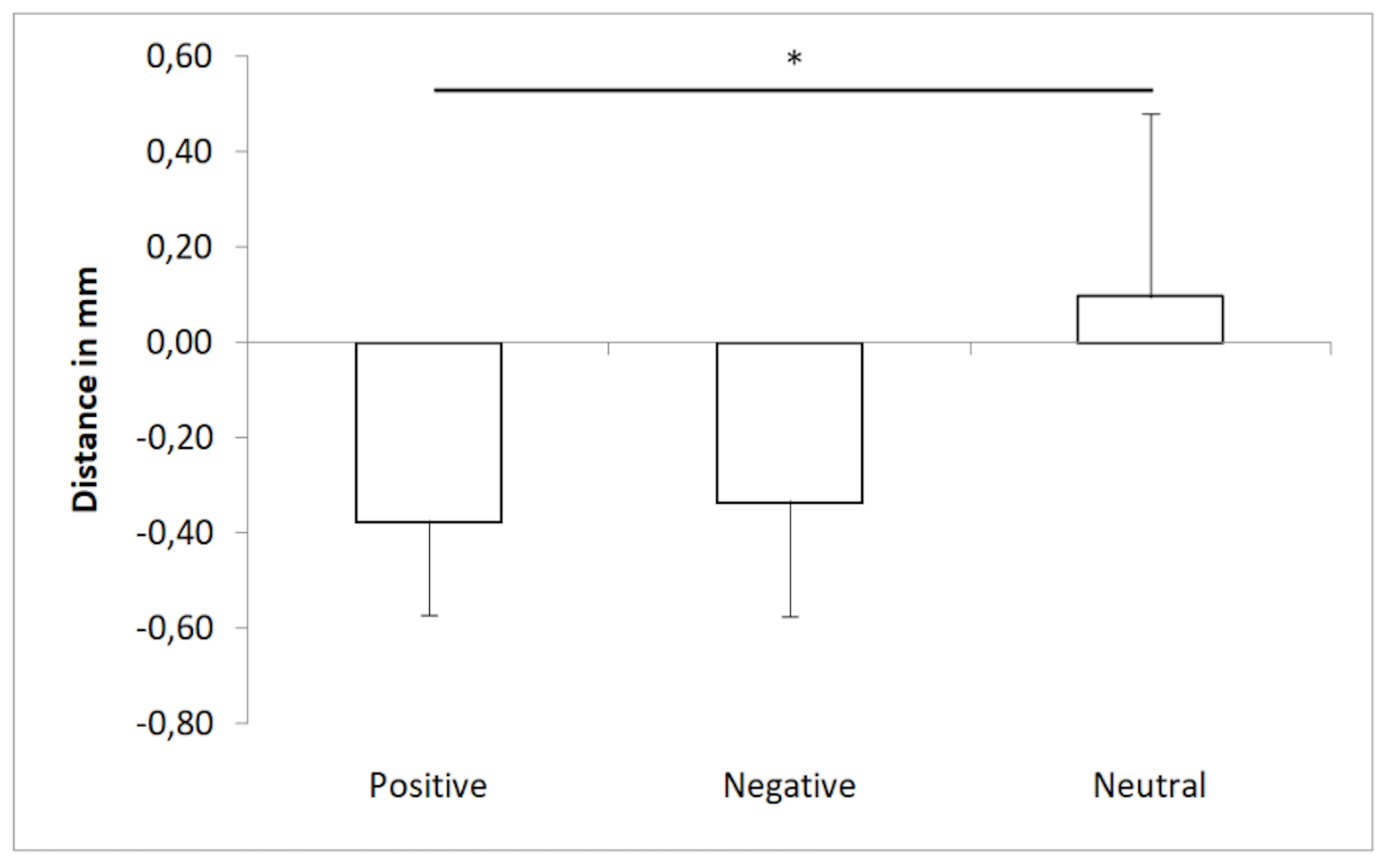
Mean COP position in anteroposterior axis according to emotion. Note. * p< .05

The mean CoP position in the AP axis was affected by emotion (*p* < 0.05): according to Tukey’s *post hoc* test (Figure 4), neutral images (0.1 ± 0.38 mm) showed more of an anterior position than positive (-0.38 ± 0.19 mm, *p* < 0.05) and negative images (-0.34 ± 0.24 mm, *p* = 0.08). The distance did not affect the mean CoP position (*p* = 0.58), and no significant difference was observed in the comparison of all distances.

## Discussion

### Subjective Evaluation

In this context, we hypothesized that the proximity of an emotional stimulus would provoke a more intense response compared to a distal stimulus. The results showed that physical distance did not influence the perception of the valence or arousal of images. These results align with the work of Mühlberger et al. (29), who stated that valence and arousal are exacerbated by proximity only for positive and neutral stimuli and not for aversive stimuli.

Davis et al. (12) asked participants to imagine spatial changes in three conditions: away, no change, or toward (i.e., perceiving the scene moving away, remaining the same, or moving closer). They showed the influence of psychological distance (imagined changes to spatial distance) on the perception of valence and arousal (emotional experience). The negative scenes were characterized by fewer negative responses and lower levels of arousal in the imagine away condition and more negative responses and higher levels of arousal in the imagine toward condition compared to the imagine no change condition.

In our study, fixed physical distance and the absence of directional movement did not present the image in a particular direction, which could explain why the perception of valence and intensity did not depend on physical distance but rather on the emotional content of the image only. Furthermore, using a three-dimensional projection, Åhs et al. (1) reproduced the two previous studies (29; 12) for a negative stimulus and obtained the same results, meaning that proximity to the stimulus exacerbates the perception of an unpleasant stimulus. They advanced results similar to our findings only for the negative image; virtual distance did not influence valence or arousal. However, these results disagree with the constructive level theory. Distal distance is considered an abstract distance, unlike proximal distance, which is considered a concrete distance in which the emotional stimulus becomes more intense (12).

Concerning valence, a pleasant stimulus often generated a higher score compared to an unpleasant or neutral stimulus, and a neutral stimulus generated a higher score compared to unpleasant content. These findings are consistent with those of Hillman et al. (17), Facchinetti et al. (14), and Naugle et al. (30), who used family and happy images as a pleasant category, mutilation images as an aversive category, and images of simple daily objects (accessories and utensils) as a neutral category. Regarding the intensity assessment, the findings confirm that the most exciting and intense images are aversive. These results are consistent with the work of Bouman and Stins (2018), Yiou et al. (45), and Stins and Beek (39). Conversely, appetitive stimuli produced fewer scores than unpleasant content and higher scores than neutral content. Our results support those of the IAPS (24).

### Objective Evaluation

#### Emotion and Posture

The existing literature has quantified an objective evaluation of the effect of emotional content on PC at a fixed distance. However, because of the contradictory results in the existing literature, it is important to verify emotion–behavior coupling. Some research has shown that the sagittal axis is influenced by valence (37; 14) or arousal (18; 7), while other research has shown that the frontal axis is influenced by valence only (3; 14). We propose that emotional content (positive or negative) could affect postural balance control more than neutral content.

In our study, and following a random projection of emotional stimuli, no significant difference in the oscillations of the CoP was observed among the three emotional contexts (positive, negative, and neutral). Gélat et al. (16) showed that the valence of an image can influence the processing of the next emotional stimulus. Indeed, their results showed that control of the CoP during a step forward was affected differently when the previous test was pleasant compared to unpleasant. This finding motivated Bouman et al. (6) to present emotional images in a blocked manner. Furthermore, Azevedo et al. (3) reported a reduction in all CoP parameters in the ML axis after 20 seconds.

It is possible that the absence of an emotional valence effect was due to the activation of the defensive system (freezing), which could be explained as an effect of the random-order image presentation; thus, presenting within a block of each emotional valence could modify the results. According to Lelard et al. (26), most recent studies have shown that the presentation of emotional stimuli induces consistent automatic responses and that emotion can alter static PC by activating defensive responses. These defensive responses can disappear if the presentation of the emotions is done by blocking in the case of positive and neutral emotions. Thus, these observations support the findings of previous work that clarified the effect of an aversive emotion on the static position. In this vein, Hillman et al. (17) showed a retreat movement in women, while men displayed small anterior postural adjustments when viewing aversive images representing attack and mutilation scenes. Azevedo et al. (3) specifically examined the effect of mutilation images on PC compared to positive or neutral images, which was characterized by an overall reduction in CoP oscillations. Facchinetti et al. (14) obtained similar results, with a reduction in PS with mutilation and affiliation images. Another possible explanation relates to the lack of forthcoming movement, which could minimize and limit the effect of the emotion. The existing literature has shown that approach movements are facilitated by positive emotions, while avoidance movements are facilitated by negative emotions (31). Flexion movements are facilitated with pleasant emotional states, while extension movements are facilitated with unpleasant emotional states (10; 13). Additionally, the perception of an emotional stimulus requires a process of internal attentional focus. Based on the work of McNevin and Wulf (28), this task could minimize CoP oscillations.

Concerning the change in the mean CoP position, the results showed an effect of arousal. The neutral images were characterized by a lower intensity than the positive and negative images. This finding is discussed in subjective evaluation, and the results are presented in Table 1 and Figure 2. Therefore, greater oscillation was observed more often with positive and negative images than with neutral images. This result is consistent with the work of Horslen and Carpenter (18), who were the first to examine the effect of emotion intensity on PS. Horslen and Carpenter (18) showed that arousal influences the frequency of CoP displacement in the AP dimension. and the electrodermal activity and anterior–posterior CoP frequency increased.

#### Distance and Posture

The main findings of this study regarding the objective assessment of CoP displacement also revealed an effect of the spatial interval that separates the participants from the emotional stimuli. Thus, the short distance of 0.5 m was characterized by a reduction in body sway compared to the other variant distances of 2.1 m, 6 m, and 10 m. Additionally, an increase in distance was accompanied by an increase in the CoP’s parameters. Thus, our results align with the work of Lê and Kapoula (25) on binocular vision, for which a distance of 40 cm reduces the surface area, standard deviation, and variance of the velocity of the CoP compared to 200 cm. The main theorists interested in the effect of distance on bipedal position (5; 33, 34, 2) have shown that an increase in distance increases bodily oscillations. The spatiotemporal displacement of the CoP at a 6-m distance was significantly different from the other distances. Based on the hypothesis that height vertigo is based on visual destabilization of free stance when the distance between the eye and the object becomes critically large, these results are consistent with those obtained by Bles et al. (5). Bles et al. (5) showed that the swaying amplitude of the body increases with the eye–object distance up to a 5-m distance, where the role of vision after this distance is highly reduced in body stabilization. The CoP parameters decrease again and become more comparable at a distance of 2.1 m. The results of our study support those of Bles et al. (5): postural instability is not linearly related to increasing distance.

#### Limits and Perspectives

First, because of the large number of female participants in this study, it would be worth carrying out an experiment with a male group to compare the results with the female group and make the generalization of the results more reliable. Second, as the effect of emotion and distance has been verified for a static position, replacing the static position with a directional movement or a precision motor task could shed light on how the central nervous system controls a variety of movement and motor skills as a function of emotion and distance. Finally, as the stimuli were presented randomly, a comparative study between the random presentation and the block presentation of emotional images is necessary to understand how the central nervous system controls posture according to each emotional valence separately. Future studies could also test the effect of the cumulative duration of each category of images on postural and movement control.

## Conclusion

The perception of valence and the arousal of emotional images depend solely on content and not on distance. The presentation of emotional images tends to activate the defensive system regardless of the emotional content, which explains the absence of a valence effect on CoP control. The distance of the emotional picture influences this PC, resulting in less amplitude for proximal distances, which could be due to the visual system. However, no effect of distance on the perception of emotions was observed. Postural instability is not linearly related to increasing distance from the eye to the object, and the perception of the emotion effect is not linked to the distance effect on PC in the static position.

### Ethics and Conflict of Interest

The author(s) declare(s) that the contents of the article are in agreement with the ethics described in http://biblio.unibe.ch/portale/elibrary/BOP/jemr/ethics.html and that there is no conflict of interest regarding the publication of this paper.
